# Effect of Surface Treatment with Er;Cr:YSSG, Nd:YAG, and CO_2_ Lasers on Repair Shear Bond Strength of a Silorane-based Composite Resin

**DOI:** 10.5681/joddd.2013.011

**Published:** 2013-05-30

**Authors:** Parnian Alizadeh Oskoee, Narmin Mohammadi, Mohammad Esmaeel Ebrahimi Chaharom, Soodabeh Kimyai, Fatemeh Pournaghi Azar, Sahand Rikhtegaran, Maryam Shojaeei

**Affiliations:** ^1^Dntal and Periodontal Research Center, Tabriz University of Medical Sciences, Tabriz, Iran; ^2^Associate Professor, Department of Operative Dentistry, Faculty of Dentistry, Tabriz University of Medical Sciences, Tabriz, Iran; ^3^Assistant Professor, Department of Operative Dentistry, Faculty of Dentistry, Tabriz University of Medical Sciences, Tabriz, Iran; ^4^Assistant Professor, Department of Operative Dentistry, Faculty of Dentistry, Qazvin University of Medical Sciences, Qazvin, Iran

**Keywords:** Composite resin, laser, repair, silorane, surface treatment

## Abstract

***Background and aims.*** The aim of the present study was to compare the effect ofsurface treatment with Er; Cr:YSSG, Nd:YAG, and CO_2_ lasers on repair shear bond strength of a silorane-based composite resin.

***Materials and methods.*** Sixty eight cylindrical samples of a silorane-based composite resin (Filtek Silorane) were pre-pared and randomly divided into 4 groups as follows: group 1: without surface treatment; groups 2, 3 and 4 with surface treatments using Er; Cr:YSSG, Nd:YAG, and CO_2_ lasers, respectively. A positive control group (group 5) was assigned in order to measure cohesive strength. Repair shear bond strength values were measured and data was analyzed using one-way ANOVA and a post hoc Tukey test at a significance level of α=0.05.

***Results.*** There were statistically significant differences in repair shear bond strength values between group 2 and other groups (P < 0.05); and between group 1and groups 3and 4 (P < 0.001); however, there were no significant differences be-tween groups 3 and 4 (P = 0.91).

***Conclusion.*** The repair shear bond strength of silorane-based composite resin was acceptable by surface treatment with lasers

## Introduction

Composite resin restorations undergo chipping, delamination, or fractures over time despite improvements in their physical and mechanical properties.^1^ Adhesive dentistry has not only paved the way for conservative restoration of carious lesions by bonding the restorative materials (composite resins) to tooth structure and minimal preparation of tooth cavities, but also it has made it possible to repair the existing restorations instead of replacing the whole restoration.^2^ The clinical performance of composite resin restorations and the effect of various factors on it depend on the properties of the polymer network^3,4^and fillers.^4,5^ These properties vary in different composite resins and are very important for the evaluation of surface treatment methods in order to repair composite resin restorations.^2^ Surface treatment of composite resins is carried out in order to remove its superficial layer, prepare a clean surface with a high level of surface energy and increase the surface area available for bonding through creation of surface irregularities.^6^ Based on a study by Brosh et al, creation of a single unit between the old and the new composite resin during the repair process is carried out through three mechanisms of chemical boding with the organic matrix, chemical bonding with the exposed filler particles and micromechanical retention.^7^

Previous studies have demonstrated the efficacy of micromechanical retention produced by diamond burs, sandblasting or acid etching in the repair bond strength of composite resins.^8,9^ Another technique for surface roughening, which has drawn attention in dentistry is the use of lasers, including Er;Cr:YSGG laser.^10,11^ Studies have shown that Erbium laser groups can influence the surface of composite resins in addition to influencing tooth surfaces. Ozel Bektas et al showed the efficacy of Er:YAG laser in surface treatment of composite resins.^12^

Kimyai et al reported that Er;Cr:YSGG can be effective in treatment of laboratory composite resin for repair purposes.^13^ In a study by Navimipour et al, surface treatment of resin-modified glass-ionomer with Er;Cr:YSGG laser increased the bond strength of composite resin to resin modified glass ionomer.^14^ In this context, CO_2_ laser with a wavelength of 10600 nm has been shown to be effective in the removal of composite resin.^15^ In addition, Chan et al showed that it is possible to remove composite resin from the enamel surface with minimal enamel damage with the use of CO_2_ laser with a wavelength of 9.3 μm.^16^ The effects of lasers on the surface of composite resins are not confined to these two laser types. Alexander et al reported that Q-switched Nd:YAG laser, too, can remove composite resin without damaging the underlying enamel.^17^ The majority of composite resins available now are based on the free radical polymerization of dimethacrylates. The main problem with these composite resins is polymerization shrinkage and the resultant stresses.^18^ Attempts have made to solve this problem, including the use of polymerization with ring-opening reaction of silorane-based molecules.^18,19^ The ring-opening reaction of silorane-based molecules is a cationic reaction during which no oxygen-inhibited layer forms.^20^ It appears that evaluation of the repair bond of silorane-based composite resin as a material with low polymerization shrinkage ^19^ is important. Therefore, the aim of the present study was to evaluate the effect of surface treatment of silorane-based composite resin with the use of Er;Cr:YSGG , Nd:YAG, and CO_2_ lasers on the repair shear bond strength of composite resin.

## Materials and Methods

Compositions and Characteristics of the composite resin and adhesive system have been presented in Table 1. Sixty-eight cylindrical samples of a silorane-based composite resin (Filtek Silorane, 3M ESPE Dental Products, St. Paul, USA) were prepared using plastic molds. The resin composite samples (with a diameter of 6 mm and a height of 4 mm) were built in increments of 2 mm. Based on manufacturer’s instructions, each layer was light-cured for 40 seconds with a light intensity of 400 mW/cm^2^ using a light-curing unit (Astralis 7, Ivoclar, Vivadent, Liechtenstein). The last layer was covered with a piece of strip matrix band and pressed with a glass slab in order to achieve a smooth surface.

**Table 1:** Characteristics and Compositions of the composite resin and adhesive system

Material	Description & Composition	Manufacturer
FiltekTM Silorane, low shrink posterior Restorative	A light curing radiopaque silorane-based composite The monomer matrix is composed of siloxane and oxirane (23% of the composition). The inorganic filler contains fine quartz particles and radiopaque yttrium fluoride (76%). Additional contents: initiator (0.9%), Stablizer (0.13%) & pigments (0.005%).	3M ESPE Dental Products, U.S.A
Filtek Silorane Bond	A filled, light –curing component bonding agent for enamel and dentin bonding. It contains a 3M ESPE hydrophobic bifunctional monomer, camphor quinine/ a silane-treated silico fillers, stabilizer.	3M ESPE Dental Products, U.S.A

Fifteen additional samples with a diameter and height of 6 mm were prepared in the same manner in order to evaluate the cohesive strength. Then the samples were embedded in acrylic resin up to a height of 2 mm and were randomly divided into 4 groups of 17 based on the surface treatment technique. In group 1, no surface treatment was carried out on the composite resin samples.

In group 2, Er;Cr:YSGG laser system (Biolaser Europe GmbH, Paintweg 10, 92685 Floss, Germany) was used with a G-type tip with a diameter of 400 μm for surface treatment. This laser system produces photons with a wavelength of 2780 nm and a frequency of 20 Hz. The output power was 3 W, the energy level was 150 mJ and the energy density was 119.42 J/cm^2^. The surfaces were treated at irradiation condition of 50% water and 60% air.

In group 3, Nd: YAG laser system (Nd:YAG Dental Laser, LAMADA Scientifica, Srl, Vicenza, Italy) was used with the fiber diameter of 400 μm for surface treatment. This laser system produces photons with a wavelength of 1064 nm and a frequency of 20Hz. The output power was as the same as group 2.

In group 4, CO_2_ surgical laser system (LAMBADA Scientifica Srl, Vicenza, Italy) was used for surface treatment. The focal point diameter of this laser system is 400 μm. This system produces photons with a wavelength of 10600 nm and a frequency of 20 Hz. The output power was as the same as group 2.

The above-mentioned conditions were determined by a pilot study and the composite surfaces were irradiated from a distance of 2 cm perpendicular to surface for 15 seconds.

After all the samples were surface treated, the surfaces were rinsed with distilled water, dried and covered with a layer of silorane bonding agent (3M ESPE, USA). After curing the bonding agent for 10 seconds and placing the plastic mold (with a length of 2 mm and a diameter of 4 mm) at the center of the samples, a new layer of composite resin with a thickness of 2 mm was placed on the surface of the previous layer and light-cured for 40 seconds using Astralis 7 light-curing unit at a light intensity of 400 mW/cm^2^. Then the plastic mold was removed and the samples were once again light-cured for 20 seconds from each direction. The samples were stored in distilled water for 24 hours at 37ºC; and then a universal testing machine (Hounsfield Test Equipment, Model H5KS, England) was used for shear bond strength test at a strain rate of 1mm/min. The force was applied by the chisel-shaped blade of the equipment at the interface of the old and new composite resin. The bond strength was measured in Newton and was converted to MPa using the following formula:

**Figure:**
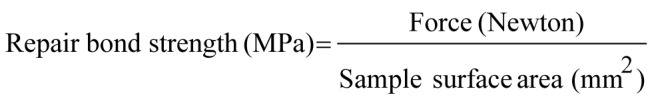


The same technique was used to measure the cohesive strength of the samples in group 5. Prior to adding new composite resin, two samples from each group were randomly selected and were gold-sputtered by a 150-Aº thin gold layer under vacuum (10^-3^ mbr); then the surface topography was evaluated under an scanning electron microscope(Tescan Vega-II ; Tescan, S.RO. Libusinia Trida, CZ) at ×1000 and kV=10. Repair shear bond strength values were analyzed by one-way ANOVA and a post hoc Tukey test. Statistical significance was defined at P<0.05.

## Results

Table 2 presents the means and standard deviations of repair shear bond strength values in the control and laser-treated groups. The highest bond strength was recorded in the Er; Cr: YSGG group (15.36±2.80) and the lowest bond strength was recorded in group1 (6.90±2.17).

**Table 2:** Means and standard deviations (SD) of repair bond strength values (MPa) in the study groups

Group	(No)	Mean±SD	Min	Max
1(No surface treated)	15	6.90±2.17	2.84	9.02
2 (Er;Cr:YSSG)	15	15.36±2.80	11.50	21.69
3(Nd:YAG)	15	11.69±2.06	8.61	14.95
4(CO_2_)	15	12.36±1.82	9.90	15.04
5(Bulk)	15	19.10±2.77	16.17	23.68

One-way ANOVA analysis revealed significant differences between the study groups (P<0.001). Two-by-two comparison of the groups with post hoc Tukey test revealed significant differences in repair bond strength between group 2 and 1 (P<0.001), group 2 and 3 (P=0.008) and group 2 and 4 (P=0.001). In addition, there were significant differences between groups 3 and 4 on the one hand and group 1 on the other (P<0.001); however, there were no significant differences between groups 3 and 4 (P=0.93).

**Figure 1:**
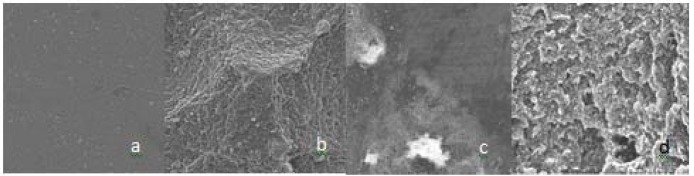
Micrographs of surface topography patterns: (a) No suface treated group; (b) Er;Cr:YSGG laser-treated group; (c) Nd:YAG laser-treated group; (d) CO_2_ laser-treated group.

Figure 1 (a-d) presents the micrographs of surface topographies in the four study groups. In the Er; Cr: YSGG group a clearly visible and homogeneous microretentive feature is seemed in the form of surface depressions. In the CO_2_ and Nd: YAG groups ablation and the surface roughness were different from those of the Er;Cr:YSGG group.

## Discussion

Silorane-based composite resins were marketed with the aim of solving the problems in relation to polymerization shrinkage, such as stresses resulting from polymerization shrinkage and water sorption.^21^ Despite a high hydrolytic stability of these materials,^22,23^ it is possible that longevity of restorations with silorane-based composite resins might be influenced by mechanical or chemical degeneration processes, which might clinically be manifested as chipping, abrasion, discoloration or recurrent caries.^24^

Successful repair of a composite resin restoration involves establishment of an appropriate interfacial bond between the previous composite resin and the new one.^25,26^ The effect of various mechanical surface treatment techniques on the repair bond strength of methacrylate-based composite resins has already been evaluated.^7,26^ Previous studies, demonstrated that the results of surface treatment in different kinds of composite resins yield different results due to differences in the structures of polymer matrices and fillers.^27,28^ According to the present situation of laser systems in treatment of tooth surfaces and restorative materials and regarding the different polymer structure of silorane-based composite resins, the aim of the present study was to evaluate the effect of surface treatment with Er;Cr:YSGG , Nd:YAG,and CO_2 _lasers on the repair shear bond strength of these materials. Based on the results, the lowest repair bond strength was recorded in the no surface treated group, consistent with the results of other studies,^13,29,30^ and indicating the important role of surface roughening and micromechanical interlocking in improving the repair bond strength of this type of composite resin.^6,9^ The highest repair bond strength was achieved in the Er; Cr: YSGG laser-irradiated group, with significant differences from the other groups. The use of Erbium lasers has previously been evaluated for the removal of cementum and composite resin restorations, in relation to their selective ablation ability.^31,32^ Ablation of composite resin by Er:YAG laser is carried out through explosive vaporization, followed by hydrodynamic ejection. During this process, rapid melting and as a result, a change in the volume of the molten material produces strong expansion forces. As a result of interaction between the forces created and the composite resin structure, projections are produced on the surface and the molten material is finally removed from the surface in the form of droplets. It has been reported that this ablation of composite resin takes place after the application of Er;Cr:YSGG laser, too.^31,33,34^ Scanning electron microscope images in the present study revealed ablation and production of pitting irregularities, without formation of the smear layer on the Er;Cr:YSGG laser-irradiated surfaces. The microretentive morphology produced on the composite resin surface increases the surface area.^13^ An increase in surface area results in an increase in the bonding surface area, modifying the distribution of stresses at the interface of the two bonded materials^29^ which finally increases repair bond strength.

In the Nd:YAG and CO_2_ laser-irradiated groups although the repair bond strength was less than that in the Er,Cr:YSGG laser-irradiated group, it was significantly higher than that in no surface treated group. It has been demonstrated that these two lasers can be used in the processing of dental materials, especially for bonding of materials to each other or bonding of materials to tooth structures. ^35^ Li et al reported that preparation of the porcelain surface with Nd: YAG laser along with light-cured composite resin produces an appropriate bond between the orthodontic bracket and porcelain.^36^ Also Poosti et al demonstrated that the effect of Nd:YAG laser on the surface of porcelain is similar to that of 9.6% fluoridric acid.^37^ In the same context, CO_2_ laser has proved successful in the bond between the orthodontic bracket and the glazed porcelain surfaces, which was attributed to the complete absorption of the CO_2_ laser wavelength.^38^ Subsequent to the absorption of laser on the porcelain surface and its conversion into heat, conchoidal tears are produced on porcelain surface,^39^ providing the mechanical retention between the composite resin and porcelain. Although there are limited studies on the effect of Nd:YAG and CO_2_ lasers on composite resin surface, Turkmen et al showed that application of Nd:YAG laser on composite resin surface results in the formation of craters, microcraks and porosities on the composite resin surface.^40^ Chan et al reported that use of CO_2_ laser is a proper technique for selective ablation of composite resin.^41^ In the present study, evaluation of scanning electron microscope images of the samples irradiated by Nd:YAG and CO_2_ lasers revealed ablation and an increase in surface roughness in a pattern different from that in the Er;Cr:YSGG laser group.

Micromorphological characteristics resulting from ablation depend on laser properties in addition to the structure of composite resin.^33^ It appears the chemical composition of silorane-based composite resin elicits different responses to these three laser types, resulting in different ablation patterns. Anyway, the microretentive properties and formation of no smear layer in the CO_2_ and Nd:YAG laser groups might justify the higher repair shear bond strength values in comparison to no surface treated group. Differences in the ablation pattern and the extent and type of surface irregularities might be the reasons for differences in the repair shear bond strength values between the laser-treated groups. It should be pointed out that in the present study the mean of repair shear bond strength value in laser-treated groups was approximately 60-70% of the cohesive strength of silorane based composite resin, which is clinically acceptable based on a study carried out by Swift et al.^42^ The differences in the repair bond strength values of composite resins in different studies might be attributed to differences in treatment protocols, aging period durations, curing methods and the type of the composite resin. Given what was discussed care should be exercised in interpretation of the results.^29,43^

Finally, it should be pointed out that it is not only difficult to compare the results with other studies but also to extend the results of in vitro studies to clinical situations; from a clinical viewpoint, aging is the result of the exposure of composite materials to the oral environment and different kinds of foods and drinks and cyclic loading in a long span of time, changing the structure of these materials.^44,45^ Therefore, it is suggested that in the future studies the repair bond strength of aged samples be especially evaluated under high C-factor conditions such as the repair of margins adjacent to tooth structure.

## Conclusion

According to the limitations of the present study it can be concluded that surface treatment of silorane-based composite resin with Er;Cr:YSGG, Nd:YAG and CO_2_ lasers provides a favorable repair shear bond strength, with the Er;Cr:YSGG laser being more effective than other lasers.
